# Unauthorized change of immunosuppressants by patients with rheumatic diseases in the COVID-19 pandemic: a cross-sectional analysis of a patient survey

**DOI:** 10.1007/s00296-023-05311-8

**Published:** 2023-03-29

**Authors:** Simon Dahm, Joerg C. Henes, Sebastian J. Saur

**Affiliations:** 1 School of Medicine, University of Tuebingen, Tübingen, Germany; 2 Centre for Interdisciplinary Clinical Immunology, Rheumatology and Auto-Inflammatory Diseases and Department of Internal Medicine II (Oncology, Hematology, Immunology, Rheumatology), University Hospital Tuebingen, Otfried-Müller-Str. 10, 72076 Tübingen, Germany

**Keywords:** Surveys and questionnaires, Cross-sectional studies, COVID-19, SARS-CoV-2, Pandemic, Rheumatic disease, Fear, Compliance

## Abstract

This study provides insight in behavior and perspective of rheumatic patients during the first COVID-19 wave. Especially, we analyzed the patients’ fear of COVID-19 and unauthorized change of immunosuppressive medication in consequence of their fear. We hereby provide data from 877 patients with valuable insights into the patients’ point of view. We retrospectively interviewed patients of our rheumatic university outpatient clinic. This way, we collected information about the patients’ personal point of view. Data like the rheumatic diagnosis and immunosuppressive medication was extracted from the health records. Statistical analysis was conducted using IBM® SPSS® Statistics (version 26). A total of 877 patients were included into our study. We could show that fear of COVID-19 was clearly present in rheumatic patients. Higher fear levels seem to be associated with comorbidity burden. Unauthorized change of immunosuppressive medication was rare in our study (5%). In our study we provide novel insight into patients’ point of view and behavior of rheumatic patients. Unauthorized change of immunosuppressive medication was rare (5%) as seen in other studies. The low rate of unauthorized change and high rate of compliance is reassuring since good disease control appears to be prognostically important in the progression of COVID-19 disease. Therefore, as the pandemic continues, treatment decisions should be made in close consultation between patient and practitioner to improve adherence and reduce morbidity and mortality.

**Supplementary Information:**

The online version contains supplementary material available at 10.1007/s00296-023-05311-8.

## Introduction

The coronavirus SARS-CoV-2 took the whole world by surprise and lead to massive containment measures as well as huge efforts in vaccine and medication development. While these efforts remain very important, this study provides insight in behavior and perspective of rheumatic patients during the first COVID-19 wave (February–May 2020) in the Southern Region of Germany. Particularly, we analyzed the patients’ fear of COVID-19 and any unauthorized change of their immunosuppressive medication in consequence of their fear. This was one of the main aims of this study since patients with rheumatic diseases often undergo immunosuppressive medication. Immunosuppressive agents impose a greater risk of infection on the patient; on the other hand, they are crucial in controlling disease activity. We hereby provide data from a cohort of 877 patients with valuable insights into the patients’ point of view.

## Methods

We retrospectively interviewed patients from our rheumatic outpatient university clinic in Tuebingen, Germany from 1st May until 17th October in 2020. Each patient who visited the clinic was asked to participate in this study. In case of agreement a questionnaire containing information about the patients’ fear levels and behavior was handed out. It was then filled out by themselves. The questionnaire was created after internal departmental discussion, in the context of our clinical experience of the COVID-19 pandemic. Unfortunately, due to the short preparation time and timeliness of the question, no pretesting, testing, or validation of the questions could be performed. The full questionnaire can be seen in the supplemental data. These data were complemented by the patients’ rheumatic diagnosis, their immunosuppressive medication such as prednisolone, conventional synthetic disease-modifying antirheumatic drugs (csDMARDs), biologics, antimalarial drugs (AMDs), Janus kinase inhibitors (JAKi), etc., from their health records. This study design was approved by the ethics committee of the medical school of “Eberhard Karls University Tuebingen” (protocol number 460/2020B0). All patients gave their written consent to the usage of their data. Inclusion criteria consisted of being 18 years or older, permission for data use, and having a rheumatic disease such as inflammatory-rheumatoid joint disease (rheumatoid arthritis + spondyloarthritis), connective tissue diseases (CTD), vasculitis, or autoinflammatory disease. Other diseases which could not be included in any of the groups above were sarcoidosis and certain immunodeficiencies.

Statistical analysis was conducted using IBM^®^ SPSS^®^ Statistics (version 26). Primarily, descriptive analysis of the collected data was performed. Additionally, we conducted a linear by linear chi-square test and a logistic regression analysis to explore an association between comorbidity and fear levels.

## Results

We included 877 patients who matched our criteria into our monocentric analysis. Most patients suffered from CTD (281 patients; 32.0%), inflammatory-rheumatoid joint diseases (266 patients; 30.3%), or vasculitis (216 patients; 24.6%). Less common were autoinflammatory diseases (92 patients; 10.5%) or other diagnoses (22 patients; 2.5%). The majority (659 patients, 75.1%) were on immunosuppressive therapy three months prior to or during the first wave of the pandemic. In terms of these 75.1%, csDMARDs were used by 50%, prednisolone by 50%, biologics by 31.9%, AMDs by 18.8%, and JAKi by 3.8% of our patients. Further characteristics are shown in Table 1. With regard to the prednisolone intake, the prednisolone dose was lower or equal to 5 mg per day (mg/d) prednisolone equivalent (PEQ) in 79,3% of all patients using prednisolone. Thus, the dose was more than 5 mg/d PEQ in 20.7% of all patients with prednisolone therapy.

**Table 1:** Characteristics of the 877 study participants

	Absolute frequency	Percentage in %
Age		
Male	314	35.8
Female	563	64.2
Rheumatologic diagnosis		
Connective tissue disease	281	32.0
Inflammatory-rheumatoid joint disease	266	30.3
Vasculitis	216	24.6
Autoinflammatory disease	92	10.5
Others	22	2.5
Immunosuppression		
Yes	659	75.1
No	218	24.9
Comorbidities (Multiple choice possible)		
Age > 60 years	240	27.4
Cardiovascular disease	189	21.6
Adiposity	163	18.6
Chronic lung disease	133	15.2
Smoking during the pandemic	116	13.2
Chronic kidney disease	54	6.2
Tumor disease	51	5.8
Diabetes	46	5.2

**Fig. 1:**
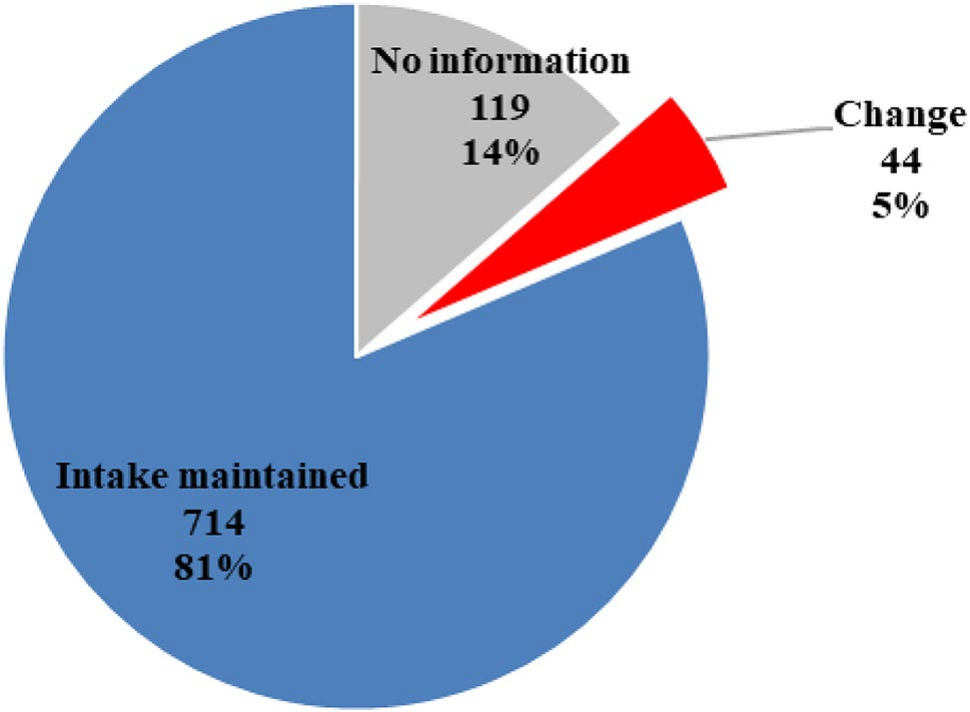
Unauthorized change of immunosuppressive medication (*n* = 877)

We asked our patients if they changed their immunosuppressive medication due to fear of a COVID-19 infection (Fig. 1). As visualized below, 714 (81.4%) patients kept on taking their medication, while 44 (5%) changed their medication without rheumatologic consultation. Among those 44 patients 36.3% (*n* = 16) reduced their dosage, 45.5% (*n* = 20) patients stopped their medication, and 18.2% (*n* = 8) tempered with their medication in a different way.

632 (72%) of the patients estimated their risk as increased. Of these 632 patients, 511 (80.9%) gave their rheumatic disease as the reason for this. The patients fear levels for a COVID-19 infection varied. 72 (8.2%) patients had very and 148 (16.9%) had high fear. Not more fear than usual was picked by 389 (44.4%) patients. 133 (15.2%) of our patients had low fear for COVID-19 and 107 (12.2%) had no fear at all. In 28 (3.2%) cases no information was given.

**Fig. 2:**
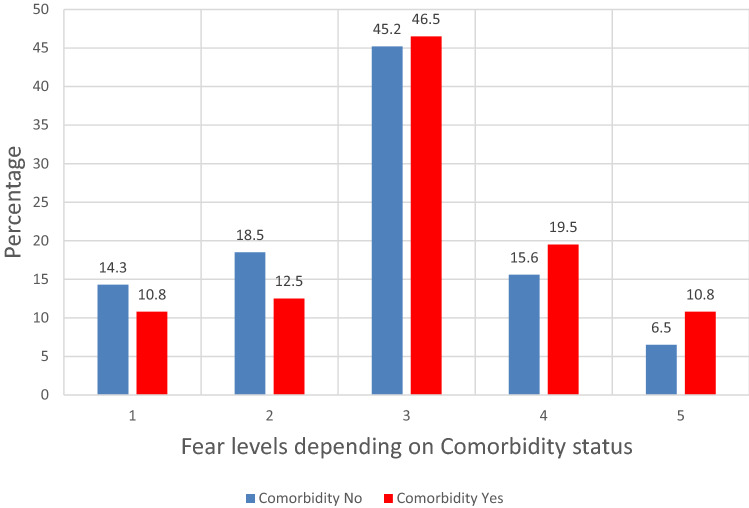
Fear levels depending on comorbidity (*n* = 849). 1: very low fear; 2: low fear; 3: not more fear than usual; 4: high fear; 5: very high fear

Figure 2 pictures the fear levels depending on the comorbidity status. Patients without comorbidities more often had lower fear levels (1 or 2), while patients with comorbidities more often had higher fear levels (4 or 5).

Furthermore, we analyzed the variables fear level and comorbidity performing a linear by linear qui square test. We calculated a p-value of 0.001 (linear by linear value: 11.728; degree of freedom: 1) which showed a significant difference with linear trend regarding patients with or without comorbidity and fear levels. We then conducted a logistic regression analysis and found patients with the highest fear level were 2.2 times more likely to have (at least) one comorbidity (odds ratio: 2.207; confidence interval: 1.20–4.06; *p* = 0.011) than patients with the lowest fear level (reference category).

## Discussion

In this cross-sectional study we revealed that patients with underlying comorbidity had a higher fear burden for COVID-19. Overall fear levels were balanced but fear burden was clearly present among our patients. Another main result was a small rate (5%) of unauthorized medication change in our cohort. Fear for COVID-19 does not seem to facilitate change of immunosuppressive medication.

In the early stage of the pandemic it was still unknown whether inflammatory rheumatic diseases or comorbidity lead to a more severe course of COVID-19 [1]. However, some risk factors for an infection in general in patients with inflammatory rheumatic diseases such as higher age, pre-existing lung disease, and diabetes mellitus were already known and listed by the German Society of Rheumatology (DGRh eV) in April 2020 [1]. Such findings might have had an impact on our patients’ fear burden.

In our study we analyzed fear levels with regard to the comorbidity status. Since in the early stage of the COVID-19 pandemic emerging data showed age to be a major risk factor for severer COVID-19 disease. Therefore, we included age > 60 years as being a comorbidity (view Table 1), so high age was incorporated into our analysis. As Table 1 shows 27, 4% (*n* = 240) of our patients, were over 60 years old. Fear levels were then evaluated but depending on comorbidity in general (Fig. 2).

We thoroughly analyzed the relationship between comorbidity status and fear for COVID-19 and found a significant difference with linear trend regarding patients with or without comorbidity and fear levels (*p* = 0.001). Further, as depicted in Fig. 2 patients without comorbidity more often had lower fear levels (1 or 2), while patients with comorbidity more often showed higher fear levels (4 or 5). In addition, a logistic regression analysis confirmed a 2.2-times higher probability for patients with the highest fear level than patients with the lowest fear level to have (at least) one comorbidity (reference category). These findings reveal that patients with comorbidities experienced higher fear burden for COVID-19.

Surprisingly, in spite of the finding above, the overall fear levels were quite balanced (low or very low fear: 27.4%; not more fear than usual: 44.4%; high or very high fear: 25.1%). However, these results show that during the first wave of the COVID-19 pandemic fear for COVID-19 was clearly present among rheumatic patients which should be taken into consideration.

Only a small part of our patients (5%) altered their immunosuppressive medication without authorization. We evaluated which medication was reduced or discontinued the most and figured out that these were methotrexate and adalimumab. One could deduce from this that especially disease-modifying drugs (DMARDs) were associated with greater fear. But since the majority of our patients suffered from rheumatoid arthritis and these drugs are very commonly prescribed in our clinic, it would be speculative to attribute an anxiety-inducing property to these drugs. Most patients adhered to their prescribed therapy. Similar results were found in a study of Fragoulis et al. where they observed a medication stop due to fear of immunosuppression in 11 (2.2%) out of 500 patients with autoimmune or rheumatic disease [2]. A similar result was seen in a study by Pineda-Sic et al. where 13 (3.8%) out of 345 patients modified or stopped their medication due to fear of COVID-19 [3]. In a German study by Schmeiser et al. patients with inflammatory rheumatic disease were asked about their personal opinion regarding their antirheumatic medication in context of COVID-19. Coherently, a majority of 90% followed the recommendation to maintain their medication. An unauthorized stop of the medication was observed in 1% of the patients and 4% said they would like to stop their medication but followed the practitioner’s advice to maintain it [4]. A study with similar focus as ours came to interesting results. Andreica et al. were confronted with a rather high rate of 20% of chronic inflammatory rheumatic disease patients who changed therapy because of COVID-19. However, after statistical examination, anxiety and disease activity were not important factors in the decision process of changing therapy [5]. The mentioned studies are especially eligible for comparison and support our findings, as they all took place during 2020 and referred to the early stage of the COVID-19 pandemic, too. To summarize, fear does not seem to increase change of immunosuppressive therapy.

The rates of medication change are also relevant since good disease control appears to be prognostically important in the progression of the COVID-19 disease. In an analysis of 6,242 patients with rheumatoid arthritis, performed by Au et al., a higher disease activity was associated with a higher probability for infection [6]. Strangfeld et al. detected a significant association between a moderate or high disease activity and a COVID-19-related death among rheumatic patients [7].

Limitations of our study were the single-center character and our data only account for the first phase of the pandemic. Due to the data collection via questionnaire some weaknesses must be taken into consideration. There were numerous Yes/No questions, so patients could have had difficulties if their answer would lie in between. Consequently, this could have led to distortion of our data. Further, we could only try to recruit patients who came into our clinic. Patients who might have had a lot of fear to go outside or were hospitalized in a different clinic due to severe COVID-19 for example, escaped our recruitment process. Comparison of all patients with rheumatoid diseases in Germany is difficult and the patients’ attitude toward COVID-19 surely changed with the ongoing pandemic. Lastly, the statistical analyzation had some weaknesses. Due to the short preparation time and timeliness of the asked question, no pretesting, testing, or validation of the questions could be performed. This would have been desirable to increase the validity of our results.

## Conclusion

In conclusion, we provide novel insight into patients’ point of view and behavior from a large cohort of 877 rheumatic patients. We could show that fear of COVID-19 was clearly present in rheumatic patients and should therefore be addressed during consultations. Higher fear levels seem to be associated with comorbidity burden. But fear does not seem to increase therapy change, because unauthorized change of immunosuppressive medication was rare in our study (5%) as seen in most other studies (2.2%, 3.8%, 1%) [2–4]. These low rates of unauthorized changes and high rates of compliance are reassuring since good disease control appears to be prognostically important in the progression of COVID-19 disease. Therefore, as the pandemic continues, treatment decisions should be made in close consultation between patient and practitioner to improve adherence and reduce morbidity and mortality.

## Supplementary Information

Below is the link to the electronic supplementary material.

Supplementary file1 (PDF 301 KB)

Supplementary file2 (DOCX 15 KB)

## Data Availability

The datasets generated during and/or analysed during the current study are available from the corresponding author on reasonable request.
