# Supplementary material for: Unauthorized change of immunosuppressants by patients with rheumatic diseases in the COVID-19 pandemic: a cross-sectional analysis of a patient survey
Source: Rheumatol Int. 2023 Mar 29;43(7):1363–7. doi: 10.1007/s00296-023-05311-8 (PMC10054195; doi:10.1007/s00296-023-05311-8)
Supplement: Supplementary file 1 — Supplementary file1 (PDF 301 KB) [file 296_2023_5311_MOESM1_ESM.pdf]

Medizinische Universitätsklinik• Otfried-Müller-Str. 10 • D-72076 Tübingen

Tübinger COVID Rheuma Befragung

Tü-CORA

Medizinische Klinik
Abteilung und Lehrstuhl II
Hämatologie
Onkologie
Klinische Immunologie
Rheumatologie

Otfried-Müller-Str. 10
72076 Tübingen

Servicetelefon:(07071) 29-84095
Fax: (07071) 29-2763
Ansprechpartner: Prof. Dr. med. Jörg Henes
Leitung Bereich Rheumatologie
Joerg.henes@med.uni-tuebingen.de

www.rheumatologie-tuebingen.de

Tübingen, den 28.09.2022

PATIENTEN-INFORMATION

Sehr geehrte Patientin, sehr geehrter Patient,

Sie leiden an einer Rheumatologischen Erkrankung und kommen regelmäßig in die Rheumasprechstunde des UKTs. Weiterhin ist nicht klar, ob Patienten mit einer rheumatologischen Erkrankung und eventuell einer rheumaspezifischen Therapie gefährdeter sind als Andere an COVID-19 zu erkranken. Wir wollen mit diesem Fragebogen evaluieren wie viele unserer Patienten an COVID-19 erkrankt sind/waren, bzw. wie viele sich infiziert /nicht infiziert haben, wenn ein naher Angehöriger nachweislich erkrankt war. Wir bitten Sie diese Daten im Rahmen einer anonymisierten Erhebung durch einen Doktoranden auswerten zu dürfen. Hierzu ist es auch wichtig zu erfragen, ob Sie ihre Rheumamedikation, selbstständig oder in Rücksprache mit ihrem Hausarzt, während der Pandemiezeit verändert bzw. gar nicht eingenommen haben.

1. Was ist der Zweck der Studie?

Der Zweck dieser Studie besteht darin, die Datenlage zur Gefahr einer Infektion mit SARS-CoV2 bei Rheumatischen Erkrankungen zu verbessern.

2. Muss ich an der Studie teilnehmen?

Die Teilnahme an der Studie ist freiwillig. Sie können die Teilnahme an der Studie jederzeit widerrufen, ohne dass Ihnen dadurch Nachteile bei der weiteren medizinischen Betreuung entstehen. Wenn Sie sich entscheiden teilzunehmen, so bitten wir Sie, eine Einverständniserklärung zu unterschreiben und den dazugehörigen Fragebogen auszufüllen. Ihre weitere Behandlung wird nicht durch Ihre Entscheidung, an der Studie teilzunehmen oder auch nicht teilzunehmen, beeinflusst.

3. Was geschieht, wenn ich teilnehme?

Wenn Sie sich entscheiden an der Erhebung teilnehmen, werden Sie gebeten den Kurz-Fragebogen zu beantworten. Neben den Fragebögen werden die Grunderkrankung sowie die Therapie zum Zeitpunkt der COVID Pandemie ausgewertet. Die Eingabe erfolgt pseudonymisiert, d.h. persönliche Daten wie Name und Adresse werden nicht in die Datenbank eingegeben. Die Daten werden pseudonymisiert gespeichert. Die Pseudonymisierungsliste wird vom Studienleiter verwaltet und die Studiendaten werden in einer speziellen, nur an der Studie beteiligten Personen zugänglichen Datei, gespeichert.

Insgesamt streben wir an, im Verlauf der nächsten 6 Monate, ca. 1000 Patienten zu befragen, um eine möglichst gute Aussagekraft zu erreichen.

Die Teilnahme an dieser Studie oder auch die Entscheidung, nicht an der Studie teilzunehmen, wird Ihre weitere medizinische Behandlung an unserem Zentrum nicht beeinflussen.

4. Was ist der Nutzen, wenn ich an dieser Studie teilnehme?

Leider gibt es für Sie keinen direkten persönlichen Nutzen. Jedoch helfen Sie die Datenlage zum Thema COVID-19 bei Rheuma zu verbessern. Dadurch wird hoffentlich in Zukunft eine bessere Beratung möglich sein.

5. Was sind die potentiellen Risiken, wenn ich an der Studie teilnehme?

Es entstehen Ihnen keine Risiken. Die diagnostischen und therapeutischen Maßnahmen weichen – bis auf die Erhebung von Daten aus vorliegenden Fragebögen - nicht von dem Standardvorgehen ab.

6. Was passiert mit den Daten?

Die erfassten Daten werden im Laufe des nächsten Jahres ausgewertet und mit bereits vorhandenen Daten aus anderen Studien verglichen und evaluiert.

7. Wird meine Teilnahme an der Studie vertraulich behandelt?

Ihre klinischen Daten werden für die Studie pseudonymisiert. Ihr betreuender Arzt klärt Sie lediglich über die Studie und die Fragebögen auf, hat aber keinen Zugriff auf die Primärdaten der Studie. Die Primärdaten aus den Fragebögen werden verblindet durch einen Doktoranden ausgewertet.

8. Ist diese Studie durch eine Ethikkommission begutachtet worden?

Die Ethikkommission der Universität Tübingen hat uns bei der Planung der Studie berufsrechtlich und berufsethisch beraten.

Vielen Dank, dass Sie diese Information durchgelesen haben.

Falls Sie Fragen haben oder mehr Information benötigen, kontaktieren Sie bitte

Prof. Dr. Jörg Henes
per e-mail: joerg.henes@med.uni-tuebingen.de oder
per Telefon: 07071/29-8 28 39
Universitätsklinik Tübingen Medizinische Klinik, Abteilung II
Otfried-Müller-Str.10
D-72076 Tübingen

Falls Sie sich entschließen, an der Studie teilzunehmen, unterschreiben Sie bitte die Einverständniserklärung.

Universitätsklinikum Tübingen

Tübinger COVID Rheuma Befragung

Tü-CORA

Tü-CORA 1.2

Medizinische Klinik
Abteilung und Lehrstuhl II
Hämatologie
Onkologie
Klinische Immunologie
Rheumatologie

Otfried-Müller-Str. 10
72076 Tübingen

Servicetelefon: (07071) 29-84095
Fax: (07071) 29-2763
Ansprechpartner: Prof. Dr. med. Jörg Henes
Leitung Bereich Rheumatologie
Joerg.henes@med.uni-tuebingen.de

www.rheumatologie-tuebingen.de

PATIENTEN-EINVERSTÄNDNISERKLÄRUNG

Datenschutzrechtliche Hinweise (EU-Datenschutzgrundverordnung)

Einwilligungserklärung zur Verarbeitung der erhobenen Daten

Information zum Umgang mit in einer Studie erhobenen Daten

Im Rahmen der Studie (*ggf. Studientitel einfügen*) werden personenbezogene Daten (Namen, Geburtstag, Adresse, Vorbefunde, studienbezogene Befunde einschließlich bildgebender Verfahren, Ergebnisse studienbezogener genetischer Untersuchungen u.a.) erhoben und verarbeitet.

In die Verarbeitung werden, soweit erforderlich, auch Ihre Krankheitsdaten einbezogen. Die Dokumentation Ihrer Daten und deren Archivierung erfolgt pseudonymisiert in einer geschützten elektronischen Datenbank, zu der nur befugte Mitarbeiterinnen und Mitarbeiter einschließlich auf das Berufs- und Datengeheimnis verpflichteter Doktorandinnen und Doktoranden Zutritt haben. Zur Überprüfung der korrekten Übertragung der Behandlungsdaten aus Ihrer Krankenakte in die verschlüsselte Studiendatenbank dürfen bevollmächtigte Personen (sogenannte Monitore) Einblick in die persönlichen Krankheitsdaten nehmen, die mit der Studie im Zusammenhang stehen. Alle beteiligten Mitarbeiter unterliegen der Schweigepflicht.

Die im Rahmen der Studie erhobenen Daten können auch für künftige Forschungsvorhaben der Klinik bzw. des Instituts genutzt und weiterverarbeitet werden.

Die Verarbeitung und Nutzung der pseudonymisierten Daten erfolgt auf Erhebungsbögen und elektronischen Datenträgern im Regelfall für die Dauer von 10 Jahren, soweit der Zweck der Studie, z. B. bei Einbringung in eine Datenbank und bei Langzeitstudien keine längere Speicherdauer erfordert.

Die im Verlauf dieser Studie gewonnenen Informationen können für wissenschaftliche Zwecke auch an Kooperationspartner im Geltungsbereich der Europäischen Datenschutz-Grundverordnung und an Kooperationspartner außerhalb des Europäischen Wirtschaftsraumes, d.h. in Länder mit geringerem Datenschutzniveau (dies gilt auch für die USA) übermittelt werden. Soweit Ihre Daten in Länder mit geringerem Datenschutzniveau übermittelt werden, wird der Verantwortliche alle erforderlichen Maßnahmen treffen, um das Datenschutzniveau zu

gewährleisten. Sollte dies nicht möglich sein, werden Ihre Daten lediglich dann übermittelt, wenn Sie in die vorgeschlagene Datenübermittlung ausdrücklich einwilligen, nachdem Sie über die für Sie bestehenden möglichen Risiken einer derartigen Datenübermittlung unterrichtet wurden.

Die Forschungsergebnisse aus der Studie werden in anonymisierter Form in Fachzeitschriften oder in wissenschaftlichen Datenbanken veröffentlicht. Bei der Veröffentlichung der Forschungsergebnisse wird Ihre Identität nicht bekannt. Die Prüfärzte vor Ort können jedoch mit Hilfe einer Patientenliste bei Rückfragen die Daten zu Ihrer Person zurückführen.

Sie können jederzeit Auskunft über Ihre gespeicherten Daten anfordern sowie die Überlassung einer kostenlosen Kopie verlangen und haben das Recht, fehlerhafte Daten berichtigen zu lassen. Sie können auch jederzeit verlangen, dass Ihre Daten gelöscht oder anonymisiert werden, so dass ein Bezug zu Ihrer Person nicht mehr hergestellt werden kann. Diese Rechte sind nach § 13 des Landesdatenschutzanpassungsgesetzes bzw. § 27 des Bundesdatenschutzanpassungsgesetzes insoweit beschränkt, als diese Rechte voraussichtlich die Verwirklichung der jeweiligen Forschungszwecke unmöglich machen oder ernsthaft beeinträchtigen und die Beschränkung für die Erfüllung der jeweiligen Forschungszwecke notwendig ist. Das Recht auf Auskunft besteht darüber hinaus nicht, wenn die Daten für Zwecke der wissenschaftlichen Forschung erforderlich sind und die Auskunftserteilung einen unverhältnismäßigen Aufwand erfordern würde.

Der Studienleiter *Prof. Dr. Jörg Henes (Rheumatologische Ambulanz des UKT, 07071-2982711)* ist für die Einhaltung der gesetzlichen Datenschutzbestimmungen im Rahmen der Studie zuständig und ist erster Ansprechpartner. Bei Beschwerden können Sie sich an den Datenschutzbeauftragten des Universitätsklinikums Tübingen oder den Landesdatenschutzbeauftragten des Landes Baden-Württemberg wenden. Für die Erhebung, Speicherung, Nutzung und Weitergabe Ihrer Daten ist Ihre ausdrückliche Zustimmung durch Unterzeichnung der Einwilligungserklärung zum Datenschutz erforderlich.

Rechtsgrundlage für die Verarbeitung Ihrer Daten sind Art. 6, 7, 9, 89 der Datenschutz-Grundverordnung in Verbindung mit §§ 4, 5, 6, 8, 9, 12, 13 des Landesdatenschutzgesetzes Baden-Württemberg in der ab 25. Mai 2018 geltenden Fassung.

Einwilligungserklärung zum Umgang mit den in einer Studie erhobenen Daten:

- **Ich erkläre, dass ich mit der im Rahmen der Studie erfolgenden Erhebung und Verarbeitung von Daten und ihrer verschlüsselten (pseudonymisierten) Weitergabe einverstanden bin.**
- **Ich stimme zu, dass bevollmächtigte Personen zum Zwecke der Überprüfung der Daten Einblick in meine persönliche Krankenakte nehmen dürfen und entbinde den behandelnden Arzt insoweit von seiner ärztlichen Schweigepflicht.**
- **Mir ist bewusst, dass die Ergebnisse dieser Studie in medizinischen Fachzeitschriften veröffentlicht werden, allerdings in anonymisierter Form, so dass ein direkter Bezug zu meiner Person nicht hergestellt werden kann.**

- **Ich wurde darüber informiert, dass ich jederzeit Auskunft über meine gespeicherten Daten und die Berichtigung von fehlerhaften Daten verlangen kann.**
- **Ich weiß, dass ich jederzeit, beispielsweise beim Widerruf der Studienteilnahme, verlangen kann, dass meine bis dahin erhobenen Daten gelöscht oder unverzüglich anonymisiert werden.**
- **Ich erkläre, dass ich über die Erhebung und Verarbeitung meiner in dieser Studie erhobenen Daten und meine Rechte angemessen informiert wurde.**
- **Ich stimme der Verwendung der im Rahmen dieser Studie erhobenen Daten in der oben (*oder in der Information zum Datenschutz*) beschriebenen Form zu.**

Kontaktinformationen:
Datenschutzbeauftragter des Universitätsklinikums Tübingen
Calwerstraße 7/4, 72076 Tübingen,
Tel. 07071 29-87667, E-Mail: dsb@med.uni-tuebingen.de

Landesbeauftragter für den Datenschutz und die Informationsfreiheit in Baden-Württemberg
Postanschrift: Postfach 10 29 32, 70025 Stuttgart
Tel.: 0711/615541-0, FAX: 0711/615541-15, E-Mail: poststelle@lfdi.bwl.de

Tübingen, den	Unterschrift	Name des Patienten/der Patientin in Blockschrift
Tübingen, den	Unterschrift	Name des aufklärenden Arztes/ Wissenschaftlers in Blockschrift

Tübinger COVID-19 Rheuma Befragung - Tü-CORA

War jemand in Ihrer engsten Familie (gleicher Haushalt) an COVID-19 erkrankt oder hatte einen positiven Rachenabstrich auf SARS-Cov2?

- ☐ Ja
- ☐ Nein

Hatten Sie sonst wissentlich Kontakt zu einem COVID-19 Erkrankten?

- ☐ Ja
- ☐ Nein

Hatten Sie während der Hochphase Februar – Mai 2020 selbst Symptome welche passend zu einer COVID-19 Erkrankungen wären?

- ☐ Nein
- ☐ Ja
  - ☐ Fieber
  - ☐ Trockener Husten
  - ☐ Muskelschmerzen oder Gelenkschmerzen
  - ☐ Verlust Geruch/Geschmack
  - ☐ Durchfall
  - ☐ Kurzatmigkeit
  - ☐ Anderes:___________________________

Wurde bei Ihnen SARS-Cov2 nachgewiesen?

- ☐ Ja
- ☐ Nein

Datum der Diagnose COVID-19: ___ ___ _______

Wurde das Virus in einem Rachenabstrich nachgewiesen?

- ☐ Ja
- ☐ Nein

Warum hatte man den Abstrich gemacht?

- ☐ Symptome passend zu COVID-19
- ☐ Kontakt zu COVID Patient

Zählen Sie sich selbst zu einem Risikopatienten im Falle einer COVID-19 Erkrankung?

- ☐ Nein
- ☐ Ja , aufgrund
  - ☐ Alter
  - ☐ Rheumaerkrankung
  - ☐ Rheumamedikation
  - ☐ Begleiterkrankungen (Diabetes, Bluthochdruck, Übergewicht)
  - ☐ Raucher
  - ☐ Sonstiges:____

Universitätsklinikum
Tübingen

Tü-CORA 1.2

Wie ist ihre aktuelle Körpergröße (in Zentimetern) und ihr Körpergewicht (in Kilogramm)?

☐ Körpergröße: _______cm
☐ Körpergewicht: _____ kg

Haben Sie Angst vor einer Infektion mit COVID 19?

☐Ja, sehr große Angst
☐Ja, große Angst
☐Nicht mehr als sonst auch
☐Wenig Angst
☐Gar keine Angst

Hatten Sie aus Angst vor einer Infektion mit SARS CoV2 selbstständig Ihre Rheumamedikation verändert?

☐ Ja , ich habe Medikamente reduziert; wenn ja welches: _____________________
☐ Ja, ich habe Medikamente gar nicht eingenommen, wenn ja welche?
____
☐ Nein, ich habe alles weiter genommen
☐ Andere eigenständige Maßnahmen:
____

Haben Sie die von uns empfohlenen Routine-Laborkontrollen beim Hausarzt in den letzten Monaten regelmäßig wahrgenommen?

☐ Ja
☐ Nein, wenn Nein, warum:

☐ Ich hatte Angst mich in der Hausarztpraxis anzustecken
☐ Ich habe keinen Termin bei meinem Hausarzt bekommen
☐ Andere Gründe: ________________________________

Wie streng haben Sie die Abstandregeln befolgt?

☐Sehr streng
☐Streng
☐Weniger streng
☐Gar nicht

Tagen Sie außerhalb der Wohnung auch in Bereichen ohne Mundschutzpflicht einen Mund-Nasen-Schutz?

☐Ja
☐ Nein

Was entspricht am ehesten Ihrem höchsten Bildungsabschluss?

☐ Ich habe die Schule nie besucht oder ohne Abschluss verlassen
☐ Hauptschule, Realschule oder abgeschlossene Lehre
☐ Allgemeine Hochschulreife/ Abitur oder Fachhochschulreife
☐ Abschluss einer Fachhochschule, Techniker- oder Meisterschule
☐ Hochschulabschluss (Magister, Diplom, Staatsexamen)

Rauchen Sie aktuell/hatten während der Corona Pandemie geraucht?

☐Ja
☐Nein

<u>Die weiteren Fragen nur ausfüllen wenn Sie an COVID-19 selbst erkrankt waren:</u>

Wie äußerte sich die Infektion bei Ihnen? (alles ankreuzen was auftrat)

- ☐ Keine Symptome
- ☐ Fieber
- ☐ Trockener Husten
- ☐ Krankheitsgefühl
- ☐ Muskelschmerzen oder Gelenkschmerzen
- ☐ Schnupfen
- ☐ Verlust Geruch/Geschmack
- ☐ Durchfall
- ☐ Kurzatmigkeit
- ☐ Anderes:___________________________

Wurden Sie stationär wegen COVID 19 behandelt?

- ☐ Ja
- ☐ Nein

Waren Sie auf einer Intensivstation?

- ☐ Ja
- ☐ Nein

Hat man ein Röntgenbild der Lunge gemacht?

- ☐ Ja
- ☐ Nein

Wurde ein CT der Lunge gemacht?

- ☐ Ja
- ☐ Nein

Zeit zwischen Auftreten der Symptome und der stationären Aufnahme: __________ Tage

Haben Sie Medikamente zur Behandlung von COVID-19 bekommen?

- ☐ Nein
- ☐ Ja:
  - ☐ Antibiotika
  - ☐ Hydroxychloroquin
  - ☐ Chloroquin
  - ☐ Tocilizumab
  - ☐ Anakinra

Haben Sie heute noch Beschwerden von der Infektion?

☐ Nein

☐ Atembeschwerden
☐ Leistungsminderung
☐ Konzentrationsstörungen

Hatten Sie einen Schub Ihrer Rheumaerkrankung im Rahmen der Infektion?

☐ Ja
☐ Nein

Vielen Dank für Ihre Mitarbeit!
