# Supplementary material for: Unauthorized change of immunosuppressants by patients with rheumatic diseases in the COVID-19 pandemic: a cross-sectional analysis of a patient survey
Source: Rheumatol Int. 2023 Mar 29;43(7):1363–7. doi: 10.1007/s00296-023-05311-8 (PMC10054195; doi:10.1007/s00296-023-05311-8)
Supplement: Supplementary file 2 — Supplementary file2 (DOCX 15 KB) [file 296_2023_5311_MOESM2_ESM.docx]

**Translation of the questionnaire from German into English:**

**Tübingen COVID-19 Rheumatism Survey - Tü-CORA**

**Has anyone in your immediate family (same household) had COVID-19 or had a positive throat swab for SARS-Cov2?**

- **Yes**
- **No**

**Have you otherwise knowingly had contact with someone with COVID-19?**

- **Yes**
- **No**

**Did you have any symptoms during the peak period of the pandemic from February - May 2020 which would be consistent with a COVID-19 disease?**

- **No**
- **Yes**
  - - **Fever**
    - **Dry cough**
    - **Muscle pain or joint pain**
    - **Loss of smell/taste**
    - **Diarrhea**
    - **Shortness of breath**
    - **Other:__________________________**

**Have you been diagnosed with SARS-Cov2?**

- **Yes**
- **No**

**Date of COVID-19 diagnosis: ___ ___ _______**

**Was the virus detected in a throat swab?**

- **Yes**
- **No**

**Why had the swab been taken?**

- **Symptoms consistent with COVID-19**
- **Contact to COVID-19 Patient**

**Do you consider yourself a patient at risk for COVID-19 disease?**

- **No**
- **Yes, due to:**
  - - **Age**
    - **Rheumatic disease**
    - **Rheumatic disease medication**
    - **Concomitant diseases (e.g. diabetes, high blood pressure, overweight)**
    - **Smoker**
    - **Other:__________________________**

**What is your current height (in centimeters) and body weight (in kilograms)?**

- **Height: _____ cm**
- **Body weight: ____ kg**

**Are you afraid of infection with COVID 19?**

- **Yes, very afraid**
- **Yes, afraid**
- **No more than usual**
- **Little afraid**
- **No afraid at all**

**Had you independently changed your rheumatic disease medication for fear of infection with SARS CoV2?**

- **Yes, I have reduced medication; if so, which: __________________**
- **Yes, I have not taken medication at all; if so which: _____________**
- **No, I have continued to take my medication**
- **Other independent actions: ________________________________**

**Have you regularly attended the routine laboratory checks recommended by us at your family doctor's office in the last few months?**

- **Yes**
- **No, because:**
  - - **I was afraid of catching an infection at my family doctor's practice**
    - **I did not get an appointment with my family doctor**
    - **Other: _________________**

**How strictly did you follow the standoff rules?**

- **Very strict**
- **Strict**
- **Less strict**
- **Not at all**

**Do you wear a face mask in areas where face masks are not required?**

- **Yes**
- **No**

**Which most closely corresponds to your highest level of education?**

- **I have never attended school or left school without a diploma**
- **Secondary school, secondary modern school or completed apprenticeship**
- **General university entrance qualification/ A-levels or entrance qualification for a university of applied sciences**
- **Graduation from a technical college, technical school or master craftsman school**
- **University degree (e.g. master's degree, diploma, state examination)**

**Do you currently/have you smoked during the Corona Pandemic?**

- **Yes**
- **No**

**Complete the following questions only if you have had COVID-19 yourself:**

**How did the infection manifest itself in you? (check all that occurred)**

- **No symptoms**
- **Fever**
- **Dry cough**
- **Feeling sick**
- **Muscle aches or joint pain**
- **Rhinitis**
- **Loss of smell/taste**
- **Diarrhea**
- **Shortness of breath**
- **Other:__________________________**

**Have you been treated as an inpatient for COVID 19?**

- **Yes**
- **No**

**Have you been in an intensive care unit?**

- **Yes**
- **No**

**Have they done an x-ray of the lungs?**

- **Yes**
- **No**

**Has a CT of the lungs been done?**

- **Yes**
- **No**

**Time between onset of symptoms and hospital admission: __________ days**

**Have you been given any medications to treat COVID-19?**

- **No**
- **Yes:**
  - - **Antibiotics**
    - **Hydroxychloroquine**
    - **Chloroquine**
    - **Tocilizumab**
    - **Anakinra**

**Do you still have symptoms from the infection today?**

- **No**
- **Respiratory problems**
- **Reduced performance**
- **Concentration problems**

**Did you have a relapse of your rheumatic disease in the context of the infection?**

- **Yes**
- **No**

**Thank you for your participation!**
